# Ambient conditions of the operating theatre and its correlation with fatigue and sleep quality of operating room workers: a cross-sectional survey

**DOI:** 10.3389/fpubh.2024.1392950

**Published:** 2024-05-15

**Authors:** Abdulmueen Alotaibi, Syed S. Habib, Thamir Al-khlaiwi, Abdulaziz Alodhayani, Zaky F. Rashed, Ghidaa Al Mahdali, Salem Alanazi, Salma Al Hassan, Saad Al-Anazi, Reem A. Al Saif, Norah A. Alsaegh

**Affiliations:** ^1^Department of Anaesthesia Technology, College of Applied Sciences, University of Almaarefa, Dariyah, Riyadh, Saudi Arabia; ^2^Physiology Department, King Saud University, Riyadh, Saudi Arabia; ^3^Department of Physiology, College of Medicine, King Saud University, Riyadh, Saudi Arabia; ^4^Health Promotion and Health Education Research Chair, College of Medicine, King Saud University, Riyadh, Saudi Arabia; ^5^Department of Anesthesia, Intensive Care and Pain Management, Faculty of Medicine, Al-Azhar University, Cairo, Egypt; ^6^Neuroscience Unit, Technical Consultant Department, Neuromodulation and Pelvic Health, Medtronic, Riyadh, Saudi Arabia; ^7^Medtronic (United States), Minneapolis, MN, United States; ^8^Security Forces Hospital, Riyadh, Saudi Arabia; ^9^Department of Physiology, College of Medicine, King Saud University, Riyadh, Saudi Arabia

**Keywords:** fatigue, staff, sleep quality, NAPS, waste anesthesia gases

## Abstract

**Background:**

Anesthesia providers face numerous occupational hazards, including exposure to anesthesia gases, which can lead to fatigue. These professionals face challenges such as night shifts, OR stress, limited mobility and sunlight access, high workload, inadequate rest breaks. Health-related sociodemographic variables, such as smoking, sleep patterns, and obesity. Our research aims to explore various risk factors associated with fatigue among operating theatre workers including sleep quality.

**Methods:**

A cross-sectional study was conducted on 227 of operating room healthcare professionals from five tertiary hospitals in Saudi Arabia, for a period of 6 months, between January 1, 2023 to June 1, 2023. The study used a five-point Likert scale sheet and the FSS “fatigue severity scale” to analyze and measure fatigue and sleep quality. The questionnaire included all socio-demographic variables, work conditions, and fatigue severity scale items.

**Results:**

The major findings revealed a significant correlation between fatigue severity scores and exposure to anesthesia gases. Socio-demographic variables such as smoking have showed major relevance to fatigue in the sample size, as (76.6%) of the participants that answered as regular smokers have showed result of positive correlation to fatigue and with a significant of (0.034). Out of the total sample, 76.1% were exposed to anesthesia gases once daily, showing a positive association with fatigue severity scores. Work-related factors like job experience and position also had a lower association with fatigue severity. *p* (0.031) Univariate logistic regression *p* (0.035).

**Conclusion:**

The study found that the work-related conditions like workload on Anesthesia technicians and technologists over 44 h per week and gas exposure is directly linked to fatigue severity and sleep quality so is the socio-demographic considerations. With poor sleep quality in younger staff which is documented in the study result a large-scale prospective analysis to understand the factors affecting OR staff’s sleep quality and fatigue severity and what can be done to regulate working hours and break time and incorporate naps in to enhance patient safety and well-being for anesthesia providers in Saudi Arabia.

## Introduction

Along with increased exposure to dangers related to chemicals, biology, ergonomics, and psychology, anesthesia providers confront a greater variety of occupational hazards (1). These threats can harm anesthesia providers’ health and affect the quality of their lives as well as their work (2). As the operating rooms are recognized to be a high-pressure environment in which anesthesia professionals labor long hours of work, and are under stress to make vital judgments and decisions within a restricted timeframe. As a result, the staff is exposed to a greater risk of fatigue and burnout (3). Up to 73.1% of anaesthesia practitioners reported that fatigue was a typical professional issue, and up to 60.8% reported that they suffered from severe excessive daytime sleepiness, these conditions can reduce attentiveness and performance, increasing the likelihood of unfavorable patient outcomes (4).

There have been several studies suggesting that exposure to anesthesia gases can adversely affect anesthesia providers’ health in that; during the surgery, nitrous oxide and volatile anesthetics are commonly employed to induce and maintain anesthesia. However, these gases can accumulate in the body repeatedly, posing a danger to anesthesia providers (5). A significant number of obstacles confront anesthesia professionals; including the strain of night shifts, unpredictable events during complex surgeries, working in closed environments with limited mobility as well as inadequate rest breaks (6). The fatigue and added stress experienced by anesthesia providers can be caused by a variety of factors. Whether ambient operating theatre conditions affect the fatigue and sleep of the providers needs to be assessed.

Compared to other medical professionals, a recent study highlighted that anesthesia professionals have been shown to experience high levels of lethargy as well as face a higher risk of occupational biological threats that can be hematogenous and aerogenic (7). Moreover, the study stated that female anesthesia providers were also found to be more burned out as they reported higher levels of exertion, lower rewards and justice, and more inadequate sleep quality (7). Patient safety has been shown to depend in part on addressing the sources of stress and exhaustion (8). For example, factors such as working in a closed environment, and limited access to sunlight which OR personnel routinely bear, contribute to fatigue (9).

Regardless of how, when it comes to the extent to which medical work descriptions and working hours influence OR healthcare personnel’s health remains unclear, especially since several types of occupational fatigue have been noticed to be common among most healthcare providers. A few studies suggested that the fatigue experienced is due to various aspects. Nevertheless, there is still a lack of knowledge regarding the specific risk factors associated with fatigue among healthcare providers in operating theaters. As a result of all the reasons mentioned previously, our research aims to explore various risk factors associated with fatigue among operating theatre workers including sleep quality.

## Materials and methods

### Study design and data selection

A total of 227 (126 males and 101 females) operating room healthcare professionals from five tertiary hospitals in Riyadh Saudi Arabia participated in this cross-sectional study. Answered completely a self-administered online questionnaire recruited participant using Convenience sampling provided by the hospital and sent through the post. The questionnaire was conducted from January 1, 2023, to June 1, 2023. The inclusion criteria were full-time or part-time anesthesia technologists/technicians, anesthesiologists, and nurses who worked in shifts allocated in adult surgery, pediatric surgery, and peripheral areas under operating theatre department management. Pregnant staff were excluded. Piloting of the questionnaire was conducted on a smaller sample prior to commencement of the study. All data of participants were kept anonymous and used only for the study. The study was ethically approved by the Institutional Review Board No: IRB23-011.

### Measures

This study used a five-point Likert scale sheet and the FSS “fatigue severity scale” which is one of the most frequently used inventories for measuring fatigue and a sleep quality scale that is simple and practical for assessment and psychometrically evaluated (10).

The details of the questionnaire are as follows:

1. Socio-demographic variables (age, gender, BMI, marital status, and social habits such as smoking).
2. Work conditions including working hours per week, exposure to sunline, time to work destination, breaks, and health-related issues.
3. Fatigue Severity Scale FSS Items. A nine-item self-report scale describing fatigue, its degree, and how it affects various tasks. Answers are graded on a seven-point scale, with (1 = being strongly disagreed and 7 = being strongly agreed). This indicates that the lowest possible score is nine, and the maximum possible score is 63 (11).
4. Sleep quality scale (SQS) items. A nine-item self-report scale describing sleep, its degree, and how it affects daily tasks (12, 13).

Answers are graded using a three-point Likert scale (1 = Poor, 2 = fair, 3 = Good). Based on scores on factors 1, 2, and 3, the ratings indicate the quality of sleep and satisfaction with it. Total scores were recorded with lower scores showing higher sleeping problems.

### Data analysis

Statistical analysis was carried out using SPSS software (IBM SPSS Statistics for Macintosh, Version 28.0) utilizing descriptive statistics to confirm the relationship between work conditions and fatigue. A *p*-value of <0.05 was considered to show a level of significance in the difference between the study samples. Categorical data is expressed in numbers and percentages (14).

Pearson Chi-square was used to assess the significant relationship between income and outcome variables. Univariate regression models were also used to evaluate the odds ratio of income variables and fatigue score and a multinomial regression model was also used for sleep assessment (15).

## Results

The study analyzed a total of 227 validated questionnaires. Table 1 presents the sociodemographic data, which indicates that (55.5%) of male participants (126) and (44.5%) of female participants (101). Marital status showed that (63.9%) were married (145) and (32.2%) were single (73). Smoking habits revealed that (67.4%) never smoked (53), (20.7%) were regular smokers (47), and (11.9%) were ex-smokers (7). Age distribution indicated that (47.1%) were below 35 years old (116), (29.5%) were between 36 and 45 years old (67), and (19.4%) were above 45 years old (44). BMI showed that (42.7%) were of normal weight (97), (35.2%) were overweight (80), and (22.1%) were obese (50). Chronic respiratory disease was reported by (9.3%) (21). Education levels included (18.9%) with a higher diploma degree (43), 46.3% with a bachelor’s degree (105), and (34.8%) with a master’s degree (79). Professions consisted of (30.8%) of anesthesia technologists (70), 37% were anesthesiologists (87), and (32%) were nurses (73).

**Table 1:** Demographics table.

Variables		*N*	(%)
Gender	Male	126	55.5
	Female	101	44.5
Marital Status	Married	145	63.9
	Single	73	32.2
	Divorced	9	4.0
Smoking	Never	153	67.4
	Regularly	47	20.7
	Ex-smoker	27	11.9
Age	<35	116	47.1
	36–45	67	29.5
	>45	44	19.4
BMI	Normal weight	97	42.7
	Overweight	80	35.2
	Obese Cat	50	22.1
chronic respiratory disease	No	206	90.7
	Yes	21	9.3
Education level	Higher Diploma	43	18.9
	Bachelor’s Degree	105	46.3
	Master, PhD	79	34.8
Profession	Anaesthesia Technologist/Technician	70	30.8
	Anesthesiologist	84	37.0
	Nurse/Operating theatre specialist	73	32.2

The findings of the participants’ demographic variables with FSS (Table 2) found a significant positive association between educational level and fatigue severity score with a *p*-value of (0.005). (72.1%, 31 individuals) held a diploma degree, (80.0%, 84 individuals) held a bachelor’s degree, and (58%, 46 individuals) held a master’s degree. This indicates that higher levels of education are linked to better fatigue severity scores among the participants. Additionally, regular smoking and fatigue severity scores had a statistically significant positive association. Out of the participants, (76.6%) who were regular smokers had higher fatigue severity scores, and this relationship was found to be significant with a *p*-value of (0.034).

**Table 2:** Association of variables with FSS (*N* = 227).

Variables		Fatigue severity score (FSS)		*p*-value
		No	Yes	
Gender	Male	34.1% (43)	65.9% (83)	0.06
	Female	22.8% (23)	77.2% (78)	
Marital Status	Married	32.4% (47)	67.6% (98)	0.236
	Single	24.7% (18)	75.3% (55)	
	Divorced	11.1% (1)	88.9% (8)	
Smoking	Never	34.0% (52)	66.0% (101)	0.034*
	Regularly	23.4% (11)	76.6% (36)	
	Ex-smoker	11.1% (3)	88.9% (24)	
Age	<35	23.3% (27)	76.7% (89)	0.080
	36–45	31.3% (21)	68.7% (46)	
	>45	40.9% (18)	59.1% (26)	
BMI	Normal weight	24.7% (24)	75.3% (73)	0.462
	Overweight	32.5% (26)	67.5% (54)	
	Obese Cat	32.0% (16)	68.0% (34)	
Chronic Respiratory Disease	No	29.1% (60)	70.9% (146)	0.957
	Yes	28.6% (6)	71.4% (15)	
Education Level	Higher Diploma	27.9% (12)	72.1% (31)	0.005*
	(Bachelor’s Degree)	20.0% (21)	80.0% (84)	
	Master, PhD	41.8% (33)	58.2% (46)	
Profession	Anaesthesia Technologist/Technician	25.7% (18)	74.3% (52)	0.063
	Anaesthesthiologist	38.1% (32)	61.9% (52)	
	Nurse	21.9% (16)	78.1% (57)	

*significant.

The findings from work-related characteristics compared to FSS (Table 3) indicate that there is a statistically significant positive relationship between job experience and fatigue severity score, with (80.6%) of participants with less than 5 years of experience reporting higher scores (*p*-value = 0.049). Similarly, there is a significant positive association between work position and fatigue severity score, with (80%) of junior staff participants reporting higher scores (*p*-value = 0.023). Additionally, working more than 44 h per week is also significantly associated with higher fatigue severity scores, with (74%) of participants working these hours reporting higher scores (*p*-value = 0.027). The study also discovered a noteworthy correlation between fatigue severity scores and exposure to anesthesia gases. Out of the (108) participants, who represented (76.1%) of the total sample, those who were exposed to anesthesia gases once daily showed a positive association with fatigue severity scores.

**Table 3:** Work-related characteristics compared to FSS.

Variables		Fatigue severity score (FSS)		*p*-value
		No	Yes	
Live Near Your Workplace	<30 min driving	30.1% (43)	69.9% (100)	0.666
	>30 min driving	27.4% (23)	72.6% (61)	
Job Experience	<5 years	19.4% (12)	80.6% (50)	0.049*
	5–10 years	25.5% (14)	74.5% (41)	
	>10 years	36.4% (40)	63.6% (70)	
Work Position	Junior Staff	20.0% (22)	80.0% (88)	0.023*
	Supervisor	30.8% (8)	69.2% (18)	
	Head and above	23.1% (3)	76.9% (10)	
	Consultant	43.2% (16)	56.8% (21)	
	Non consultant	29.1% (66)	70.9% (161)	
Working Hours Per Week	36–40 Hours	55.0% (11)	45.0% (9)	0.027*
	40–44 Hours	27.6% (21)	72.4% (55)	
	>44 Hours	26.0% (34)	74.0% (97)	
Last Month’s Working Area	Operating Theatre Pediatric Procedures	37.9% (11)	62.1% (18)	0.361
	Operating Theatre Adult Procedures	27.0% (50)	73.0% (135)	
	Peripheral Areas	38.5% (5)	61.5% (8)	
Break At Least 30 Min	Very rarely	38.0% (19)	62.0% (31)	0.222
	Some times	24.5% (26)	75.5% (80)	
	Most of the time	29.6% (21)	70.4% (50)	
Anesthesia Gases Exposure	Never	46.9% (15)	53.1% (17)	0.031*
	Once daily	23.9% (34)	76.1% (108)	
	Once weekly	32.1% (17)	32.1% (36)	
Sunlight Exposure	No	25.7% (39)	74.3% (113)	0.073
	Yes	36% (27)	64% (48)	

*significant.

The demographic data associated with sleep as presented in Table 4, indicate a significant positive correlation between age and sleep score. Among the participants, the majority (45.5%) who were over 45 years old had a good sleep score. Additionally, (25%) had a fair sleep score, while 29.5% had a poor sleep score. The (*p*-value = 0.010) further confirms the statistical significance of this relationship. A positive correlation was also discovered between marital status and sleep scores, the majority of participants were married, with (4) individuals representing (37.2%) having a good sleep score, (47) individuals representing (32.4%) having a fair sleep score, and (44) individuals representing (30.3%) having a poor sleep score. This relationship was found to be statistically significant, with a (*p*-value = 0.009). Furthermore, a positive and statistically significant relationship exists between sleep scores and educational level. Among employees with a bachelor’s degree, (40.0%) had a poor sleep score, (35.2%) had a fair sleep score, and (24.8%) had a good sleep score (*p*-value = 0.019).

**Table 4:** Demographic characteristics associated with sleep (*N* = 227).

Variables		Sleep score			*p*-value
		Poor	Fair	Good	
Age	<25	36.2% (36)	44.0% (47)	19.8% (43)	0.010*
	36–45	32.8% (41)	29.9% (35)	37.3% (25)	
	>45	29.5% (77)	25.0% (82)	45.5% (68)	
BMI	Normal weight	37.1% (36)	35.1% (34)	27.8% (27)	0.796
	Overweight	30.0% (32)	40.0% (40)	30.0% (24)	
	Obese weight	34.0% (17)	32.0% (16)	34.0% (17)	
Gender	Male	28.6%(36)	37.3%(47)	34.1% (43)	0.126
	Female	40.6% (41)	34.7% (35)	24.8% (25)	
Marital Status	Married	30.3% (44)	32.4% (47)	37.2% (54)	0.009*
	Single	37.0% (27)	45.2% (33)	17.8% (13)	
	Divorced	66.7% (6)	22.2% (2)	11.1% (1)	
Education Level	Higher Diploma	44.2% (19)	32.6% (14)	23.3% (10)	0.019*
	(Bachelor’s Degree)	40.0% (42)	35.2% (37)	24.8% (26)	
	Master, PhD	20.3% (16)	39.2% (31)	40.5% (32)	
Chronic Respiratory Disease	No	34.0% (70)	35.9% (74)	30.1% (62)	0.979
	Yes	33.3% (7)	38.1% (8)	28.6% (6)	
Profession	Anaesthesia Technologist/Technician	38.6%(27)	37.1% (26)	24.3% (17)	0.116
	Anaesthesthiologist	23.8% (20)	38.1% (32)	38.1% (32)	
	Nurse	41.1% (30)	32.9% (24)	26.0% (19)	
Smoking	Never	34.6% (53)	31.4% (48)	43% (52)	0.135
	Regularly	29.8% (14)	51.1% (24)	19.1% (9)	
	Ex-smoker	37% (10)	37% (10)	25.9% (7)	

Data is presented as number and percent. *significant.

Table 5 showed a statistically significant positive correlation between living near workplaces and sleep scores (*p*-value = 0.026). Among the participants who commuted for less than 1:30 h, (35.7%) had good sleep scores, (30.8%) had fair sleep scores, and (33.6%) had poor sleep scores. On the other hand, among those who commuted for more than 1:30 h, (20.2%) had good sleep scores, (45.2%) had fair sleep scores, and (34.5%) had poor sleep scores.

**Table 5:** Work-related characteristics compared to sleep (*N* = 227).

Variables		Sleep score			*p*-value
		Poor	Fair	Good	
Sunlight Exposure	No	37.5% (57)	34.2% (52)	28.3% (43)	0.268
	Yes	37.5% (20)	34.2% (30)	28.3% (25)	
Live Near Your Work-Place	<1:30 h driving	33.6% (48)	30.8% (44)	35.7% (51)	0.026*
	>1:30 h driving	34.5% (29)	45.2% (38)	20.2% (51)	
Job Experience	<5 years	33.9% (21)	45.2% (28)	21.0% (13)	0.218
	5–10 years	38.2% (21)	34.5% (19)	27.3% (15)	
	>10 years	31.8% (35)	31.8% (35)	36.4% (40)	
Work Position	Junior Staff	35.5% (39)	38.2% (42)	26.4% (29)	0.882
	Supervisor	42.3% (11)	30.8% (8)	26.9% (7)	
	Head and above	30.8% (4)	38.5% (5)	30.8% (4)	
	Consultant	27.0% (10)	32.4% (12)	40.5% (15)	
	Non consultant	31.7% (13)	36.6% (15)	31.7% (13)	
Working Hours Per Week	36–40 Hours	20.0% (4)	55.0% (11)	25.0% (5)	0.443
	40–44 Hours	35.5% (27)	35.5% (27)	28.9% (22)	
	>44 Hours	35.1% (46)	33.6% (44)	31.3% (41)	
Last Month’s Working Area:	Operating Theatre Paediatric Procedures	37.9% (11)	34.5% (10)	27.6% (8)	0.072
	Operating Theatre Adult Procedures	32.4% (60)	38.9% (72)	28.6% (53)	
	Peripheral Areas	46.2% (6)	0.0% (0)	53.8% (7)	
Break (At Least 30 Min)	Very rarely	46.0% (23)	26.0% (13)	28.0% (14)	0.056*
	Sometimes	34.0% (36)	41.5% (44)	24.5% (26)	
	Most of the time	25.4% (18)	35.2% (25)	39.4% (28)	
Anaesthesia Gases Exposure	Never	25.0% (8)	43.8% (14)	31.3% (10)	0.074
	Once daily	40.8% (58)	32.4% (46)	26.8% (38)	
	Once weekly	20.8% (11)	41.5% (22)	37.7% (20)	

Data is presented as number and percent. *significant.

Furthermore, about another positive correlation, it was found that participants who took breaks of at least 30 min exhibited varying sleep scores. Out of the (106) participants, (16) individuals (24.5%) had a good sleep score, (44) participants (41.5%) had a fair sleep score, and (36) participants (34.0%) had a poor sleep score. It is worth noting that these findings yielded a (*p*-value = 0.056).

Table 6 shows the results of the univariate logistic regression, indicating several statistically significant positive relationships with fatigue severity. Firstly, there is a significant positive relationship between educational level and fatigue severity (*p* = 0.007, OR = 1.548, 95% CL = 0.682–3.516) for participants with a bachelor’s degree. Additionally, job experience (*p* = 0.053, OR = 1.713, 95% CL = nurses) and work position (*p* = 0.027, OR = 0.563, 95% CL = 0.216–1.462) also show positive statistically significant relationships. Furthermore, working hours per week (>44 h, *p* = 0.037, OR = 3.487, 95% CL = 1.330–9.140) and anesthesia gas exposure (*p* = 0.035) are significantly associated with fatigue severity.

**Table 6:** Association of demographic variables with Fatigue severity (*N* = 227).

Univariate					
Variable	Subcategory	*N*	OR	95% CI	*p*-value
Age	<25	287	Ref.	–	0.084
	36–45	32	0.665	0.339–1.302	
	>45	92	0.438	0.209–0.918	
Marital status	Married	234	Ref.		0.258
	Single	24	1.465	0.776–2.767	
	divorced	58	3.837	0.466–31.575	
Educational level	Higher Diploma	299	Ref.	–	0.007*
	Bachelor	29	1.548	0.682–3.516	
	Post-grad	36	0.540	0.242–1.204	
BMI	Normal weight	97	Ref.	–	0.463
	Overweight	80	0.683	0.354–1.317	
	Obese Cat	50	0.699	0.329–1.482	
Gender	Male	126	Ref.	–	0.063
	Female	101	1.757	0.971–3.180	
Chronic respiratory disease	No	206	Ref.	–	0.957
	Yes	21	1.027	0.380–2.774	
Sunlight Exposure	No	152	Ref.	–	0.108
	Yes	75	0.614	0.338–1.113	
live near your workplace	<30 min driving	143	Ref.	–	0.667
	>30 min driving	84	1.140	0.627–2.074	
Profession	Anesthesia-related	154	Ref	–	0.104
	Nurse	73	1.713	0.895–3.278	
Job Experience	<5 years	62	Ref.	–	0.053*
	5–10 years	55	0.703	0.293–1.685	
	>10 years	110	0.420	0.200–0.880	
Work position	Junior Staff	110	Ref.	–	0.027*
	Supervisor	26	0.563	0.216–1.462	
	Head and above	13	0.833	0.211–3.287	
	Consultant	37	0.328	0.147–0.731	
	Non consultant	41	0.353	0.162–0.768	
Working hours per week	36–40 Hours	20	Ref.	–	0.037*
	40–44 Hours	76	3.201	1.161–8.827	
	>44 Hours	131	3.487	1.330–9.140	
Last month’s working area	Operating Theatre Paediatric Procedures	29	Ref.	–	0.366
	Operating Theatre Adult Procedures	185	1.650	0.729–3.786	
	Peripheral Areas	13	0.978	0.255–3.756	
Break (at least 30 min)	Very rarely	50	Ref.	–	0.227
	Sometimes	106	1.886	0.916–3.884	
	Most of the time	71	1.459	0.679–3,137	
Anesthesia gases exposure	Never	32	Ref.	–	0.035*
	Once daily	142	2.803	1.267–6.201	
	Once weekly	53	1.869	0.758–4.608	

Results from Univariate logistic regression. Data is presented as number and percent. *significant.

The results from the multinomial model of with demographics and work related variables compared to sleep in Table 7 indicate a significant association between breaks of at least 30 min and poor sleep (*p*-value ≤0.035). Participants who rarely had breaks were 3.7 times more likely to experience poor sleep, while those who sometimes had breaks were 3.2 times more likely to have poor sleep. Additionally, the education level also showed a significant association with poor sleep, with a *p*-value of (0.028). Specifically, participants with higher diploma degrees had an odds ratio of (7.116 and, a 95% confidence interval: of 1.240–40.853) compared to other education levels.

**Table 7:** Association of variables with poor\good sleep (*N* = 227).

Reference Category	Sleep	Variable	Subcategory	*N*	Multinomial		
					OR	95% CI	*p*-value
Good:	Poor	Gender:	Male	126	0.492	0.148–1.639	0.248
			Female	101	Ref.	–	
		Marital status:	Married	145	0.083	0.006–1.253	0.072
			Single	73	0.157	0.010–2.520	
			divorced	9	Ref.	-	
		Smoking:	Never	153	0.514	0.129–2.048	0.346
			Regularly	47	1.089	0.214–5.530	
			Ex-smoker	27	Ref.	–	
		Live near your workplace	<0.30 h driving	143	1.308	0.041–3.512	0.595
			>0.30 h driving	84	Ref.	–	
		Profession	Anaesthesia Technologist/Technician	70	0.774	0.134–4.461	0.775
			Anaesthesthiologist	84	0.631	0.068–5.833	
			Nurse	73	Ref.	–	
		Education level	Associate Degree (Higher Diploma)	43	7.116	1.240–40.853	0.028*
			(Bachelor’s Degree)	105	5.391	0.964–30.151	
			Postgraduate (Master, PhD)	79	Ref.	–	
		Job Experience	<5 years	62	0.620	0.098–3.933	0.612
			5–10 years	55	1.320	0.360–4.842	
			>10 years	110	Ref.	–	
		Work position	Junior Staff	110	0.322	0.049–2.127	0.239
			Supervisor	26	0.573	0.074–4.423	
			Head and above	13	0.674	0.051–8.865	
			Consultant	37	2.335	0.507–10.758	
			Non-Consultant	41	Ref.	–	
		Working hours per week	36–40 Hours	20	0.214	0.034–1.336	0.099
			40–44 Hours	76	0.976	0.389–2.449	
			>44 Hours	131	Ref.	–	
		Last month’s working area	Operating Theatre Pediatric Procedures	29	1.857	0.279–12.361	0.522
			Operating Theatre Adult Procedures	185	1.323	0.272–6.438	
			Peripheral Areas	13	Ref.	–	
	
		Break for at least 30 min.	Very rarely	50	3.743	1.101–12.728	0.035*
			Sometimes	106	3.220	1.147–9.038	
			Most of the time	71	Ref.	–	
		anesthesia gases exposure	Never	32	1.482	0.295–7.435	0.632
			Once daily	142	2.740	0.953–7.883	
			Once weekly	53	Ref.	–	
		Age	<25	116	3.632	0.716–18.421	0.120
			36–45	67	1.259	0.338–4.694	
			>45	44	Ref.	–	
		BMI	Normal weight	97	0.593	0.173–2.034	0.406
			Overweight	80	0.676	0.214–2.133	
			Obese	50	Ref.	–	
		Sunlight Exposure	No	152	1.419	0.542–3,718	0.476
			Yes	75	Ref.	–	
chronic respiratory disease	No	206	0.775	0.176–3.413	0.736

Results from nominal multivariant regression.

## Discussion

In this study, we aimed to investigate factors contributing to fatigue, with a specific focus on exposure to waste anesthesia gases. An intriguing and distinctive observation in our research study was the significant association between anesthesia gas exposure among operating theatre staff and Fatigue Severity Scale (FSS) scores. Notably, this association was particularly pronounced in the subgroup of individuals who reported daily exposure to anesthesia gases, constituting 62.5% of our study sample. This key finding highlights an increase in the frequency of smelling anesthetic gases among operating theatre staff.

Moreover, our findings align with a study conducted in the USA, where a substantial group of Certified Registered Nurse Anesthetists was surveyed regarding their occupational exposure to inhalational anesthetic agents, yielding quite similar results (5). The study demonstrated that exposure to waste anesthesia gases contributes to fatigue (9, 17). Anesthetic gas pollution can arise from various sources, including anesthesia breathing circuits like pediatric T-piece circuits, especially in the absence of a proper scavenging system and the maintenance of the operating theatre ventilation system (5).

Our study also showed a significant correlation between education levels and FSS. Bachelor’s and associate degrees had higher levels of FSS which may correlate to the higher workload. We found that nurses’ high level of fatigue which is not concomitant with the findings of Nurses’ level of education operating room had not correlated to job fatigue (17, 18) which makes the Anesthesia technician, and technologist in our opinion possible target of this association. Furthermore, smoking in this study shows a significant correlation with FSS. The majority of the previous operating room staff studies did not include smoking data or they had a smaller percentage of smokers and were not significantly apparent (17). However, our study is consistent with (19, 20).

Work-related characteristics including Job Experience and work position have shown a significantly lower association compared to FSS. Education, experience, and years of employment did not significantly explain the resilience in OR nurses (18), Moreover, working hours per week significantly show a higher association with FSS, and severity is found among workers over 44 h which is expected due to higher strain among nursing and anesthesia technology due to shortage of staff, lack of department support, and lack of independence in planning and organizing (21) In addition, from author perspective, Anesthesia technologist looking for extra hours’ payment due to lower income which increases the workload and prevalence of FSS.

Our demographic data show that among education level groups, a Bachelor’s significantly associated with poor sleep which may explain the minimum fatigue scores mentioned previously. However, our nominal multivariant regression data compared to poor sleep affirm this association and showed a five times high association among associate and bachelor’s degree O.R staff. Fatigue among the young generation may be explained by to social habits of Saudis to sleep in average less than 6 h (22).

Our related work data showed significantly poor sleep is more prevalent in 46.0% of staff groups who rarely take a break during shift hours. Scheduled naps during long shifts improved awareness, reduced fatigue, and improved sleep quality in nurses (23, 24). Nevertheless, our nominal multivariant regression analysis significantly comparing the frequent staff group to other groups showed a progressive association, the fewer breaks group had poorer sleep quality which warrants for measures to ensure regular breaks during shift adherence.

### Strength & limitations

We conducted a detailed well-designed questionnaire as well as reliable methodological measurement. As well as an organized and detailed questionnaire looking at different environmental factors affecting operating theatre workers. Possible limitations of our study are a limited number of sample size and its cross-sectional design. Moreover, Smokers are limited and they represent 20% of our sample, unable to do vitamin screening or dehydration in addition, we were unable to collect random samples of waste anesthesia gases to compare the workers’ exposure to international recommended exposure limits. However, the targeted hospitals were tertiary and accredited by local entities, Saudi Central Board for accreditation of Healthcare Institutions CBAHI and Joint Commission International JCI were they recommend to have adequate waste Anesthesia Gas active scavenging systems and efficient positive ventilation systems with minimum air exchanges per hour interval (16, 25).

## Conclusions and recommendations

Our results showed a significantly work load in workers over 44 h per week correlated with fatigue severity in addition to Bachelor’s and associate degrees’ Anesthesia technician, and technologist staff showing poor sleep quality in overall younger staff.

A progressive significant association was shown with, less breaks staff groups with poorer sleep quality. Work-related characteristics including Job Experience and work position has shown a significantly lower association compared to FSS.

We recommend that large-scale prospective studies are needed to unravel the factors associated with work-related factors in OR staff. Operating theatre staff workload need to be regulated to limit working hours to less than 44 h per week and staff should regularly take their break time with the possibility to consider naps.

Anesthesia gases exposure among operating theatre staff had significantly shown a high association with FSS. The weight of evidence regarding potential health risks from exposure to anesthetic agents in environments suggests that clinicians need to be concerned and warrant further studies that randomly sample higher exposure areas in the operating theatres for maximum recommended limits to improve the sleep quality and reduce fatigue severity, and to confirm the causality of different environmental variables with a large number of departments and operating theatre participants.

## Data availability statement

The original contributions presented in the study are included in the article/supplementary material, further inquiries can be directed to the corresponding author.

## Ethics statement

The studies involving humans were approved by Approved by AlMaarefa University Institutional Review Board No: IRB23-011. The studies were conducted in accordance with the local legislation and institutional requirements. The participants provided their written informed consent to participate in this study.

## Author contributions

AmA: Writing – original draft, Writing – review & editing, Investigation. SH: Supervision, Writing – original draft. TA-k: Conceptualization, Data curation, Formal analysis, Funding acquisition, Investigation, Methodology, Project administration, Resources, Software, Supervision, Validation, Visualization, Writing – review & editing. AaA: Conceptualization, Data curation, Formal analysis, Funding acquisition, Investigation, Methodology, Project administration, Resources, Software, Supervision, Validation, Visualization, Writing – review & editing. ZR: Conceptualization, Data curation, Formal analysis, Funding acquisition, Investigation, Methodology, Project administration, Resources, Software, Supervision, Validation, Visualization, Writing – review & editing. GM: Writing – original draft, Writing – review & editing. SA: Conceptualization, Writing – review & editing. SH: Investigation, Methodology, Writing – review & editing. SA-A: Software, Visualization, Writing – review & editing. RS: Validation, Visualization, Writing – review & editing. NA: Validation, Visualization, Writing – review & editing.

## References

[1] Hui L Garnett A Oleynikov C Boamah SA. Compassion fatigue in healthcare providers during the COVID-19 pandemic: a scoping review protocol. BMJ Open. (2023) 13:e069843. doi: 10.1136/bmjopen-2022-069843, PMID: 37258070 PMC10255032

[2] Keller S Yule S Smink DS Zagarese V Safford S Parker SH. Episodes of strain experienced in the operating room: impact of the type of surgery, the profession and the phase of the operation. BMC Surg. (2020) 20:e937. doi: 10.1186/s12893-020-00937-y PMC7720529 33287776

[3] Irandoost SF Yoosefi Lebni J Soofizad G Chaboksavar F Khalili S Mehedi N. The prevalence of burnout and its relationship with capital types among university staff in Tehran, Iran: a cross-sectional study. Heliyon. (2021) 7:e06055. doi: 10.1016/j.heliyon.2021.e06055, PMID: 33553742 PMC7848643

[4] Scholliers A Cornelis S Tosi M Opsomer T Shaproski D Vanlersberghe C. Impact of fatigue on anaesthesia providers: a scoping review. Br J Anaesth. (2023) 130:622–35. doi: 10.1016/j.bja.2022.12.011, PMID: 36697276

[5] Masselink T Hardinger J Bowman-Dalley C O’Guinn C Hendrix K Crowell N. Certified registered nurse anesthetists’ occupational exposure to inhalational anesthetic agents: a survey of anesthetic gas safety. BMC Anesthesiol. (2022) 22:375. doi: 10.1186/s12871-022-01896-y, PMID: 36463138 PMC9719248

[6] Teymoori E Zareiyan A Babajani-Vafsi S Laripour R. Viewpoint of operating room nurses about factors associated with the occupational burnout: a qualitative study. Front Psychol. (2022) 13:947189. doi: 10.3389/fpsyg.2022.947189, PMID: 36033007 PMC9403988

[7] Magnavita N Soave PM Ricciardi W Antonelli M. Occupational stress and mental health among anesthetists during the COVID-19 pandemic. Int J Environ Res Public Health. (2020) 17:18245. doi: 10.3390/ijerph17218245, PMID: 33171618 PMC7664621

[8] Mansour A Waleed Riad AM. The occupational fatigue in anesthesiologists: illusion or real? Middle East J Anaesthesiol. (2010) 20:529–34. 20394249

[9] Golvani J Roos L Henricson M. Operating room nurses’ experiences of limited access to daylight in the workplace—a qualitative interview study. BMC Nurs. (2021) 20:227. doi: 10.1186/s12912-021-00751-8, PMID: 34753467 PMC8579627

[10] Yi H Shin K Shin C. Development of the sleep quality scale. J Sleep Res. (2006) 15:309–16. doi: 10.1111/j.1365-2869.2006.00544.x 16911033

[11] Sharma S Sheth M. Psychometric properties of Gujarati version of fatigue severity scale (FSS). J Soc Indian Physiother. (2019) 3:34–7. doi: 10.18231/j.jsip.2019.003

[12] Zheng G Li K Wang Y. The effects of high-temperature weather on human sleep quality and appetite. Int J Environ Res Public Health. (2019) 16:20270. doi: 10.3390/ijerph16020270 PMC6351950 30669302

[13] Shahid A Wilkinson K Marcu S Shapiro CM. STOP, THAT and one hundred other sleep scales. (2012); 1–406

[14] IBM. IBM SPSS software [internet]. Ibm, (2018). 1. Available at: https://www.ibm.com/analytics/data-science/predictive-analytics/spss-statistical-software

[15] Hayes A. Chi-square (*χ*^2^) statistic: what it is, examples, how and when to use the test. Investopedia. (2022):1–10.

[16] CBAHI. Cbahi Standards. Cbahi. (2022), Available at: http://cbahi.securehostsite.biz/arabic/home

[17] Erdoğan C Doğan S Çakmak R Kizilaslan D Hizarci B Karaaslan P. Assessment of job satisfaction, work-related strain, and perceived stress in nurses working in different departments in the same hospital: a survey study. Ain Shams J Anesthesiol. (2020) 12:1–10. doi: 10.1186/s42077-020-00084-9

[18] Akansel N Akansel M Yanik H. Association of organisational stress with fatigue in operating room nurses. Int J Caring Sci. (2019) 12:627–38.

[19] Katz P Margaretten M Trupin L Schmajuk G Yazdany J Yelin E. Role of sleep disturbance, depression, obesity, and physical inactivity in fatigue in rheumatoid arthritis. Arthritis Care Res. (2016) 68:81–90. doi: 10.1002/acr.22577, PMID: 25779719 PMC6083443

[20] Kahraman T Ozdogar AT Abasiyanik Z Ozakbas S. Associations between smoking and walking, fatigue, depression, and health-related quality of life in persons with multiple sclerosis. Acta Neurol Psychiatr Belg. (2021) 121:1199–206. doi: 10.1007/s13760-020-01341-2, PMID: 32222910

[21] Almodibeg B Smith H. The prevalence and the most significant sources of occupational burnout syndrome amongst anesthetic technicians in Saudi Arabia: a cross-sectional survey. Saudi J Anaesth. (2021) 15:149–54. doi: 10.4103/sja.sja_1220_20, PMID: 34188633 PMC8191275

[22] Al-Tannir MA Kobrosly SY Al-Badr AH Salloum NA Altannir YM Sakkijha HM. Characterizing sleeping habits and disturbances among Saudi adults. Saudi Med J. (2016) 37:1372–80. doi: 10.15537/smj.2016.12.17373 27874154 PMC5303777

[23] Han K Hwang H Lim E Jung M Lee J Lee S. Scheduled naps improve drowsiness and quality of nursing care among 12-hour shift nurses. Int J Environ Res Public Health. (2021) 18:1–11. doi: 10.3390/ijerph18030891 PMC7908576 33498593

[24] Oriyama S. Effects of 90- and 30-min naps or a 120-min nap on alertness and performance: reanalysis of an existing pilot study. Sci Rep. (2023) 13:9862. doi: 10.1038/s41598-023-37061-9 37332041 PMC10277286

[25] JCI. Safety checklists protecting those who serve. (2020), Available at: https://www.jointcommission.org

